# A Prosthetic and Surgical Approach for Full-Arch Rehabilitation in Atrophic Maxilla Previously Affected by Peri-Implantitis

**DOI:** 10.1155/2021/6637500

**Published:** 2021-03-31

**Authors:** Angélica Letícia Reis Pavanelli, Erica Dorigatti de Avila, Luiz Antônio Borelli Barros-Filho, Francisco de Assis Mollo Junior, Joni Augusto Cirelli, Luiz Antônio Borelli Barros, Rafael Scaf de Molon

**Affiliations:** ^1^Department of Diagnosis and Surgery, São Paulo State University (UNESP), School of Dentistry, Araraquara Sao Paulo, Brazil; ^2^Department of Dental Materials and Prosthodontics, São Paulo State University (UNESP), School of Dentistry, Araraquara Sao Paulo, Brazil; ^3^Department of Social Dentistry, São Paulo State University (UNESP), School of Dentistry, Araraquara Sao Paulo, Brazil

## Abstract

Rehabilitation of atrophic maxilla with dental implants is still a challenge in clinical practice especially in cases of alveolar bone resorption due to peri-implantitis and pneumatization of the maxillary sinuses. Several surgical approaches have been employed to reconstruct the lost tissues allowing the proper tridimensional position of the implants. In this context, the aim of this case report is to describe a surgical and prosthetic approach to fully rehabilitate the atrophic maxilla with dental implants. The patient presented with unsatisfactory functional and esthetical implant-supported prosthesis with some of the implants already lost by peri-implantitis. The remaining three implants were also affected by peri-implantitis. Reversal prosthetic planning was performed, and a provisional prosthesis was fabricated and anchored in two short implants. Sinus floor augmentation procedure and onlay bone graft were then accomplished. After a healing period of 8 months, digital-guided surgery approach was performed to place the implants. Finally, a definitive prosthesis was installed. One-year follow-up has revealed stabilization of the bone tissue level, successful osseointegration, and a pleasant esthetic and functional result. A proper diagnosis and careful planning play an important role to enhance precision and to achieve patient esthetic and functional outcomes.

## 1. Introduction

Dental implants have been extensively used to rehabilitate partial or total edentulous patients in the recent decades [1, 2]. To obtain appropriate implant retention, it is essential to have sufficient amount of bone volume in the receptor area, and in many cases, this condition is not suitable, for instance, in cases of long-term tooth loss leading to alveolar bone atrophy, bone defects due to different types of trauma, and pneumatization of the maxillary sinuses [3]. All of those issues impair the installation of dental implants in a proper tridimensional position [2, 4].

When teeth are lost due to periodontal disease, dental cavities, or trauma and the oral rehabilitation is not performed in a reasonable amount of time, the alveolar process tends to resorb. In this regard, the reduction in bone density, resorption of the alveolar ridge, and pneumatization of the maxillary sinuses are characteristics that usually affect patients that have lost their teeth for a long time [5, 6]. In this context, several approaches have been used to rehabilitate atrophic areas in the maxilla with dental implants. Among the various techniques used for rehabilitation of atrophic jaw, some of them can be highlighted: guided bone regeneration with rhBMP-2 [7–9]; osteogenesis by alveolar distraction [10, 11]; autogenous, homogenous, and xenogenous bone graft [7–9, 12–14]; bone reconstruction with vascularized free flap [15]; and the use of short dental implants (dental implants with less than 8 mm in length) [2, 16–18]. The use of autogenous bone graft is still the gold standard to rehabilitate atrophic jaws due to its osteogenic, osteoinductive, and osteoconductive properties [19]. However, in cases of severe atrophy of the maxilla, the amount of bone graft necessary to fully reconstruct the area is quite high, which requires an extra oral donor area such as the iliac crest region [20] or calvarial bone graft [21, 22]. Autogenous bone graft has some drawbacks, for instance, limited amount of bone graft that can be harvested (intraoral approach), increased surgical time and rehabilitation, high costs, intensive postsurgical care, and excessive morbidity to the patient especially when extraoral graft is harvested.

Trying to overcome the disadvantages of autogenous bone graft, guided bone regeneration with bone substitute materials and growth factors (recombinant human bone morphogenetic protein-2 (rhBMP-2), plasma-rich platelets (PRP), and leukocyte- and platelet-rich fibrin (L-PRF)) have been proposed and tested [23, 24]. Although positive results have been demonstrated with guided bone regeneration and rhBMP-2 [25, 26], the elevated costs of rhBMP-2 (infuse bone graft), healing time, surgical morbidity, and risk of membrane and biomaterial exposure decrease patient acceptance. On the other hand, L-PRF seems to allow early placement of dental implant in maxillary sinuses [27] and increased bone formation when combined with demineralized bovine bone mineral (DBBM) [28]. In order to minimize patient discomfort, morbidity, costs, and healing time, bone substitute materials such as demineralized bovine bone mineral (DBBM) have been extensively used to fill maxillary sinuses in atrophic upper jaw with high success rate and low morbidity to the patient [29, 30]. This approach permits implant placement with a sufficient length allowing proper osseointegration and anchorage of the implant. This approach is aimed at reducing comorbidities and allowing faster implant rehabilitation with minimal complications to the patient.

Finally, implant design, implant abutment connections (implant platform), and physical-chemical surface characteristics play pivotal role in the osseointegration process enhancement (implant microtopography) and in primary implant stability (macrotopography) especially in patients requiring immediate implant loading and in patients with poor bone quality and quantity [31–33]. Thus, this case report is aimed at presenting a complex case of oral rehabilitation with dental implants in a patient with severe atrophy of the posterior maxilla, associated with the presence of some implants with peri-implantitis. The clinical management of this case is fully presented, and the chosen approaches are therefore discussed.

## 2. Case Report

**Figure 1:**
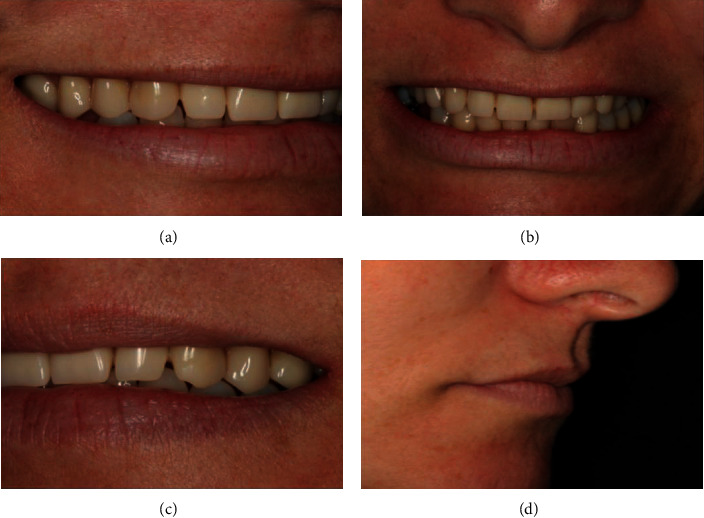
Clinical images of the patient's smile.

**Figure 2:**
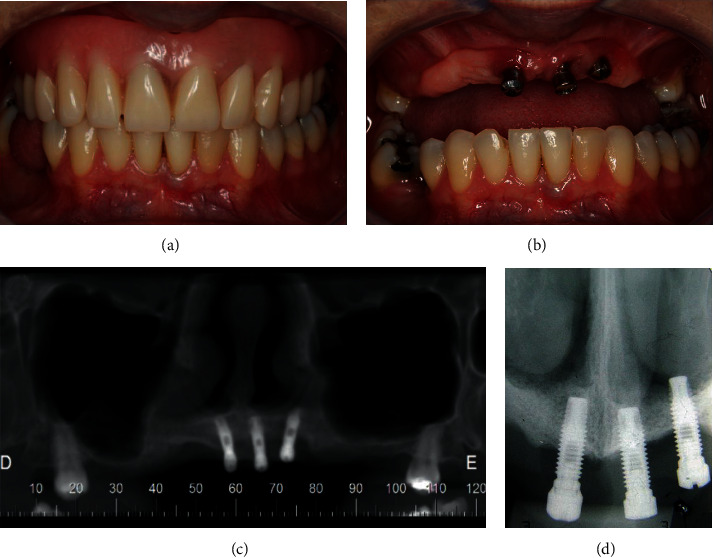
Intraoral images of the prosthesis in placement (a). After prosthesis removal, it is possible to observe gingival inflammation and swelling of the implants (b). Panoramic and periapical radiographies show evident bone resorption around the implant threads and severe pneumatization of the maxillary sinuses (c, d).

A 45-year-old Caucasian female patient sought for dental treatment in the Department of Periodontology with the main complaints of dissatisfaction with the use of a total upper denture (Figures 1(a)–1(d)). Patient medical records were not significant, and she denied use of alcohol or smoking. Her clinical history was marked by premature tooth loss in her adult age and by the use of removable prosthesis thereafter. She underwent implant rehabilitation in the maxilla eight years ago, and some implants were lost due to peri-implantitis in the posterior area of the maxilla. Clinical and radiographic examination revealed poor oral hygiene and the presence of three dental implants in the anterior area with gingival bleeding, swelling, and localized calculus in the implant surface (Figures 2(a) and 2(b)). Panoramic radiography showed extensive pneumatization of the maxillary sinuses, a radiolucent area around the remaining implant threads, and alveolar bone loss around the implant threads near the apical third of the fixation (Figures 2(c) and 2(d)).

Based on her medical history and clinical and radiographic analyses, the treatment plan was proposed in different stages. The first treatment phase encompassed new provisional prosthesis, surgical removal of the implants affected by peri-implantitis followed by the installation of two short implants with immediate loading, and provisional prosthesis installation. In the second phase, the surgical approaches included maxillary sinus floor augmentation bilaterally with concomitant graft with DBBM plus PRF membranes, autogenous block bone graft, implant placement, and definitive prosthetic rehabilitation. The planned surgical reconstruction in different phases of the treatment was intended to allow the patient to use provisional prosthesis during her treatment without functional and esthetic complaints. Written informed consent was obtained prior to initial treatment.

**Figure 3:**
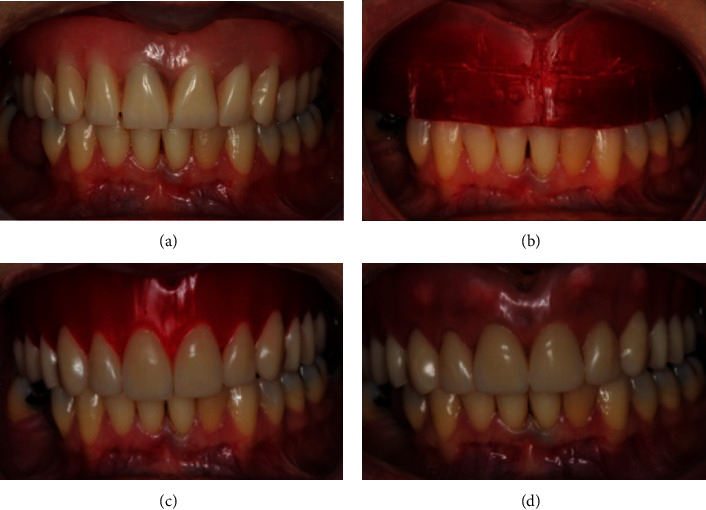
Prosthetic procedures were carried out to install a provisional prosthesis attached to two short implants by means of o-ring connection (a–d).

**Figure 4:**
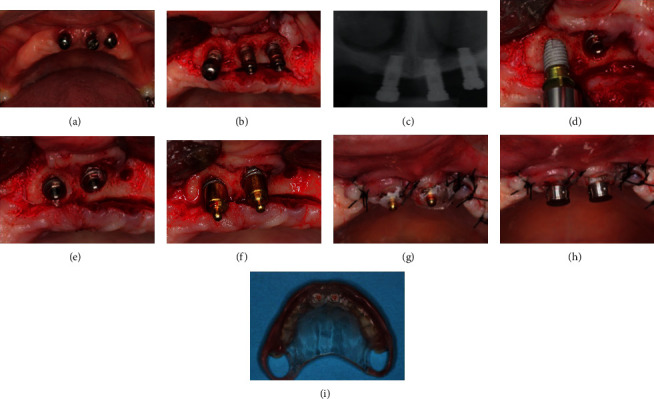
The first step in the surgical procedures was to expose the area where the implants were installed, and then, the implants were removed (a–c). After implant removal, two short implants were immediately installed. An appropriate abutment was installed in each of the implants to allow provisional prosthesis installation with appropriate retention (d–i).

The first step of treatment involved the confection of new provisional prosthesis previous to implant removal. In this moment, all the expectations of the patient regarding her tooth characteristics (color, size, position, etc.) and all the necessary changes in her prosthesis were taken into account to achieve all the patient's functional and esthetic requirements. The prosthetic procedures are represented by Figures 3(a)–3(d). Subsequently, two remote vertical releasing incisions were performed in the alveolar crest followed by a crestal incision on the area where the implants were installed (Figures 4(a) and 4(b)). Periapical radiography was taken to evaluate the implants (Figure 4(c)). A full-thickness flap was elevated on the buccal and palatal aspects. The removal of the implants was performed with the aid of a trephine drill trying to preserve the remaining alveolar bone as much as possible. Then, two short implants were installed in the remaining alveolar bone crest (Figures 4(d)–4(f)) (Conexao® Sistema de Protese, external hexagons, 5.0 × 5.5 and 4.3 × 5.5 mm). The implant insertion torque for both implants was higher as 45 Ncm, which permitted the immediate function of the implants. The prosthetic abutment connection was installed, and the soft tissue was closed by means of simple suture (Figures 4(g) and 4(h)). The o-ring connection was able to assist the retention of the provisional total prosthesis (Figure 4(i)).

**Figure 5:**
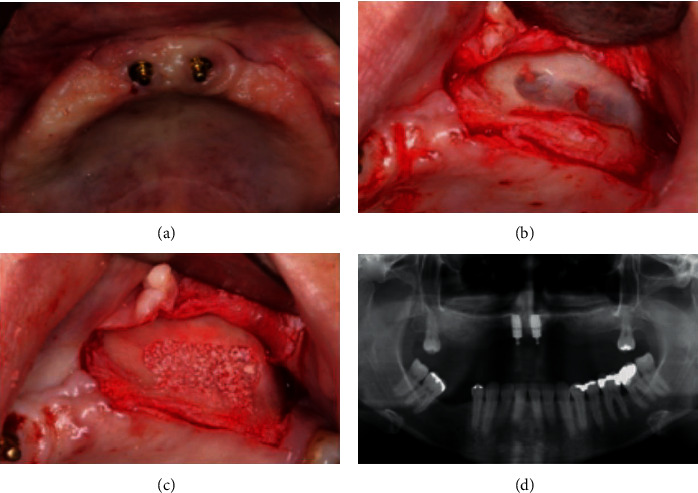
The second surgical procedure was carried out to augment the maxillary sinuses. DBBM plus L-PRF was used to fill the sinuses aiming at increasing bone height to allow proper implant length installation (a–c). Panoramic radiography shows the maxillary sinuses immediately after bone graft (d).

The next step in the process of rehabilitation was to perform the maxillary sinus floor augmentation. This procedure was completed four months after short implant placement. The surgical procedure consisted by a midcrestal and vertical releasing incisions alongside the remaining alveolar bone to reveal the lateral sinus wall [28]. A lateral window approach was performed to access the sinus wall using diamond round bur. The surgical access respected the position of implant placement planning and the maxillary sinus anatomy [27, 28]. After careful sinus membrane elevation, the maxillary sinuses were filled with a mixture of L-PRF and large particles [29, 30] (1-2 mm) of DBBM (Bio-Oss, Geistlich Pharma AG, Wolhusen, Switzerland). Membranes of L-PRF were also used to cover the lateral window of the sinus cavity. The soft tissue was then sutured with absorbable sutures (Vicryl, Ethicon, Somerville, NJ, USA). After the surgical procedure, a panoramic radiography was taken to evaluate the maxillary sinus bone gain (Figure 5(d)).

**Figure 6:**
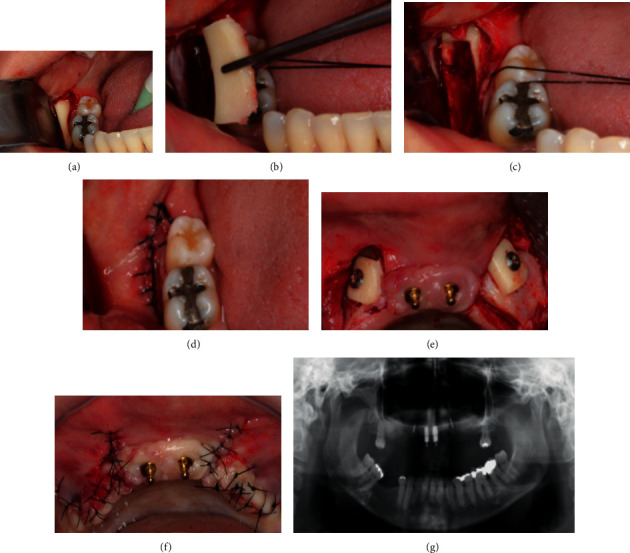
The third surgical approach was to install an onlay autogenous block bone graft harvested from the mandibular ramus. The graft was split and was installed in the receptor area in the posterior maxilla to augment the alveolar crest width and consequently to allow implant installation in an ideal tridimensional position (a–f). Panoramic radiography was taken immediately after graft placement (g).

After four months of the maxillary sinus augmentation procedure, it was planned to increase the bone thickness in the maxillary anterior area with autogenous block bone graft. The autogenous bone graft was harvested from the mandibular ramus. For this, an access preparation was carried out with a crestal and vertical releasing incision in the retromolar region. Then, a mucoperiosteal flap was obtained by exposing the buccal wall and the oblique line. The osteotomy was performed with rotating instruments and the cortical block was obtained with a hammer and chisel, as described [34]. The mucoperiosteal flap was repositioned, and the defect was sutured with Vicryl 4-0 thread (Figures 6(a)–6(d)). Thereafter, the autogenous bone was split in two parts and then were placed in position in the receptor area and fixed with cortical screws (Figures 6(e) and 6(f)). Panoramic radiography was taken immediately after graft placement (Figure 6(g)). The patient was instructed to apply chlorhexidine (0.12%) twice a day. Ibuprofen 600 mg three times a day for 3 days was prescribed for pain relief and amoxicillin 875 mg plus 125 mg of potassium clavulanate three times a day for 7 days. The area healed uneventfully, and sutures were removed at two weeks. The patient reported minimal swelling and discomfort postoperatively.

**Figure 7:**
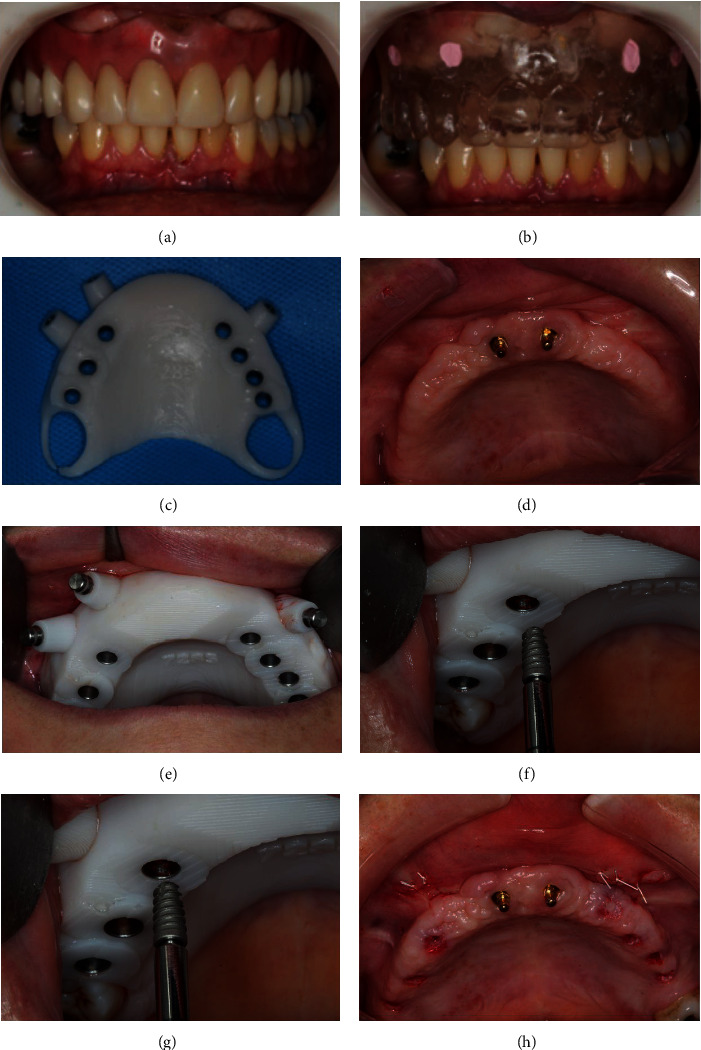
After a healing period of 8 months from the maxillary sinus augmentation procedure, the implants were installed by means of guided surgery. Seven implants were planned and installed in the posterior area of the maxilla (a–h).

The implants were placed with the aid of guided surgical approach after a 4-month onlay graft healing. Initially, the provisional prosthesis was filled with proper acrylic material (due to the internal prosthesis wear performed after graft placement) and was adjusted to allow precise adaptation to the augmented area, with ideal occlusion, fitting, and esthetics. The prosthesis was then duplicated to be used as multifunctional guide. Four vestibular gutta-percha fillings assisted as radiographic markers. A silicone interocclusal record was made to maintain the denture in a stable interarch position, during the cone beam computed tomography (CBCT) scan. A cone beam technique (I-CAT Cone Beam; Hatfield, PA, USA) was used to scan the patient. A second scan was made of the prosthesis extraorally. Virtual planning was performed with seven implants in the posterior area of the maxilla (three in the right side), as described [35]. Three guided anchor pins were planned to fix the surgical guide. A 3D planning was made with the aid of computerized images. The diameter, length, and positioning of the implants were defined, and the files were sent to Bioparts (Brasilia, DF, Brazil) to manufacture the surgical templates (Figures 7(a)–7(c)). The surgical template was stabilized with three screws, before the instrumentation for implant installation. The Cone Morse Due implants (Implacil De Bortoli, Sao Paulo, SP, Brazil) with 3.5 mm in diameter and varied lengths were used to rehabilitate the patient. After implant installation, the cover screws were installed to all implants (due to low insertion torque) and a healing period of 3 months was set to tissue healing (Figures 7(d)–7(h)).

**Figure 8:**
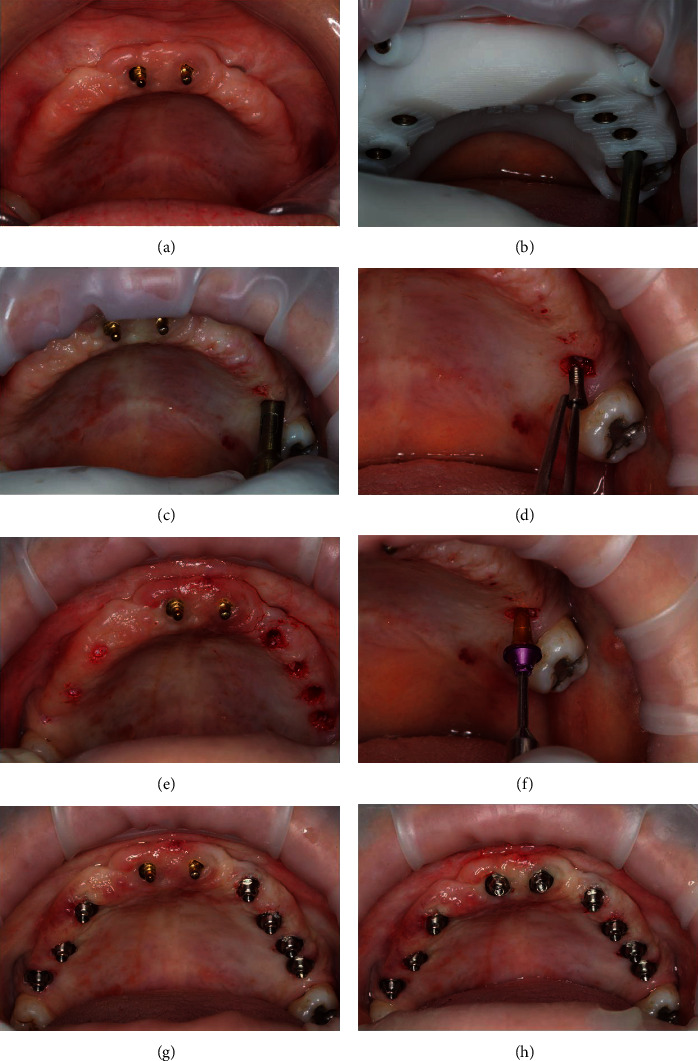
The implants were allowed to heal for 4 months, and then, the procedures to expose the implants were initiated (a, b). The cover screws of all implants were removed with the aid of the surgical template (c–f). Appropriate abutments were chosen and installed (g, h).

**Figure 9:**
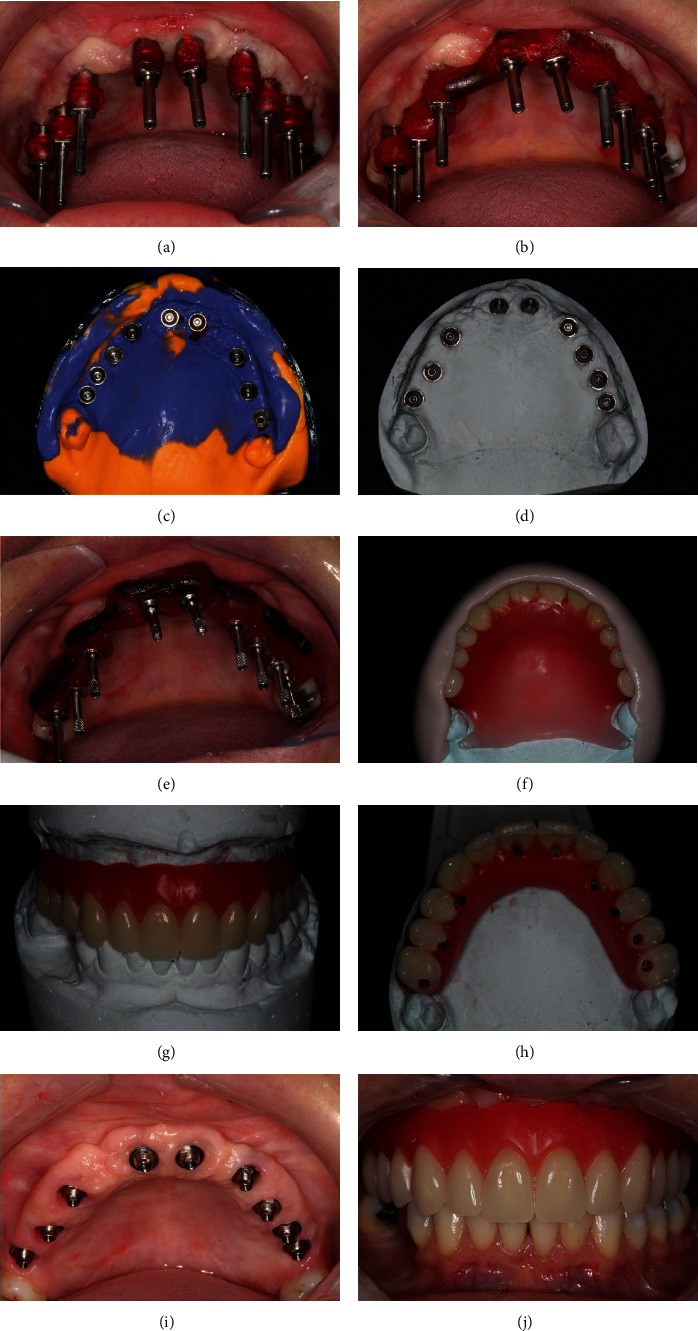
Prosthetic procedures to manufacture the definitive prosthesis were performed. All the necessary steps to create the definitive prosthesis were carried out (a–j).

**Figure 10:**
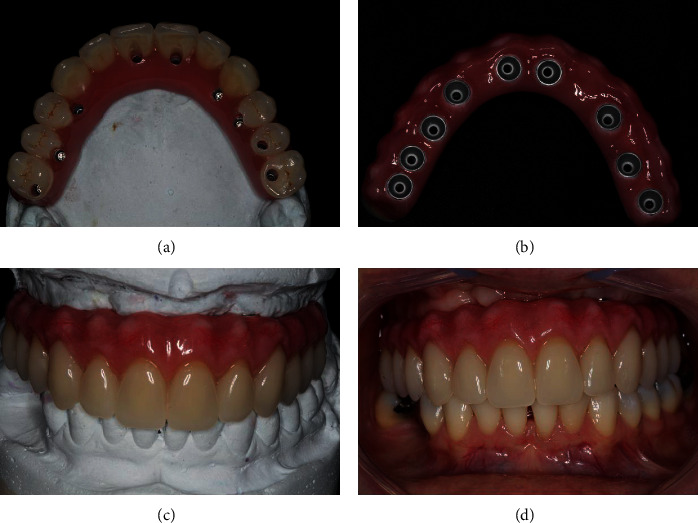
The definitive prosthesis was installed.

**Figure 11:**
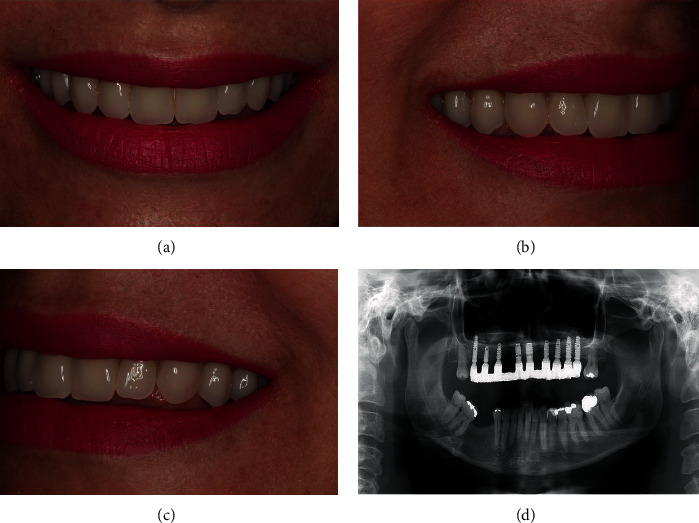
(a–c) One-year follow-up shows the successful functional and esthetic outcomes and proper osseointegration of the implants without any signs of infection.

After the healing period of 3 months, the implants were exposed and the cover screws were removed (Figures 8(a)–8(d)). A miniconical abutment was chosen for all implants, and the mini pillars were then installed (Figures 8(e)–8(h)). Finally, definitive prosthesis was carried out following all the necessary steps of prosthesis fabrication (Figures 9(a)–9(j)). The definitive prosthesis was installed in the patient (Figures 10(a)–10(d)). After one year of follow-up, the patient was satisfied with the final result in relation to the esthetics and function (Figures 11(a)–11(c)). Panoramic radiography was taken after one year to show the implants without any signs of infection and proper osseointegration (Figure 11(d)).

## 3. Discussion

Rehabilitation of maxillary deficiencies with dental implants can be a challenge for most of the clinicians and surgeons, and in many instances, a multidisciplinary approach with the involvement of several disciplines is mandatory to achieve satisfactory functional and esthetic outcomes. The increased expectation for a pleasant esthetic result from the patient is something that increases the challenge for a successful oral rehabilitation. In this context, the advancements in tissue engineering, bone regeneration, bone substitute biomaterials, implant surface, and design have promoted the development of new materials and techniques for the successful treatment of complex cases. Moreover, the introduction of new technologies, such as cone beam computed tomography, guided implant surgery, and new software, allow the achievement of more predictable and safe surgical procedures. Although interdisciplinary treatments usually result in better clinical outcomes, the increased treatment duration, costs, and miscommunication between clinicians should be taken into account when planning a complex oral rehabilitation.

In the case presented, it was chosen to perform the treatment in several steps. The initial treatment plan, i.e., the confection of provisional prosthesis supported by two short implants, was selected to allow the proper rehabilitation of the patient without affecting the masticatory functions, esthetics, and social behavioral. As the previous prosthesis was not adequate in terms of esthetics and functions due to the loss of the implants by peri-implantitis [36], the prosthesis was disregarded and a new one was confectioned. The placement of two short implants in the anterior region was capable to support the prosthesis with the o-ring connection. This surgical and prosthetic treatment also allowed us to evaluate the patient smile, lip support, phonetics, tooth size, and satisfaction with the new implant-supported prosthesis. This approach called reverse planning is indicated in pretty much all cases because the patient can adapt and accept the new clinical condition before the installation of the definitive prosthesis.

The placement of conventional length implants (>10 mm) in the posterior maxilla in the presence of insufficient bone height usually requires sinus floor augmentation procedures. Several studies in the literature have demonstrated successful results with dental implants in maxillary sinus grafted with autogenous, bone substitute materials, and combinations thereof. Thus, after a healing period of 4 months, it was decided to perform the sinus floor augmentation procedure using a combination of DBBM and L-PRF. This approach was chosen because previous studies have demonstrated beneficial effects when the maxillary sinus is graft with combination of DBBM and growth factor, allowing early implant placement and more new natural bone formation [27, 28, 37, 38]. The rationale behind the use of growth factor in maxillary sinus is to provide a stable scaffold when mixing with the DBBM and also to increase the process of angiogenesis and osteogenesis. The healing period after maxillary sinus augmentation was set as 8 months to allow proper maturation of the bone.

During the follow-up visits, it was decided to graft the anterior alveolar crest with an onlay autogenous bone graft to increase the width of the alveolar bone. This surgical procedure is aimed at correcting horizontal deficiencies in the maxillary area allowing the installation of implants in an ideal tridimensional position. According to Buser et al. [39], an improper implant positioning might lead to esthetic and functional complications and should be avoided to achieve satisfactory outcomes. Therefore, the autogenous bone was harvested from the mandibular ramus, placed in position with titanium screws, and the area was let to heal for 4 months. The rationale to perform the surgical procedures for bone augmentation (in maxillary sinus and alveolar crest) in two different time points is to decrease patient comorbidities and discomfort. Moreover, as the healing period for maxillary sinus augmentation with DBBM takes longer than autogenous bone graft, the treatment duration was not affected.

After the healing period, the placement of implants was planned by means of computed guided surgery. After CBCT scan of the patient and denture, the virtual planning was performed and the files were sent to Bioparts (Brasilia, DF, Brazil) to manufacture the surgical templates. The conical implants were installed with the flapless approach, which facilitates would healing and decreased postoperative pain [35]. Virtual planning and flapless approach are very reliable and accurate treatment modalities and present some advantages over the conventional implant surgery, such as correct implant positioning, high level of accuracy, faster implant placement, faster wound healing, better treatment planning, and less amount of anesthetics necessary. As the implants were installed over the bone graft and the primary stability of the implants were lower than 40 Ncm, it was decided to not load the implants immediately after surgery. Three months were waited before the definitive prosthesis was confectioned. Finally, the prosthetic procedures were comprehensively performed and the definitive prosthesis was installed.

Despite the fact that the present treatment approaches have led to a satisfactory functional and esthetic result, it also presents some disadvantages that need to be taken into account. The virtual planning of the implants is time-consuming and comes with a higher cost due to multiple CBCT scans and template fabrication. This method necessitates good communication with the dental technician. Moreover, implant graft (maxillary sinus and onlay autogenous bone) increases the treatment duration and costs and might lead to postoperative discomfort.

## 4. Conclusions

In summary, this case report illustrates an efficient surgical-prosthetic approach for the management of a complex case of peri-implantitis, which required surgical reconstruction of the maxillary bone, dental implants, and prosthetic rehabilitation. A proper diagnosis and careful planning play an important role to enhance precision and to achieve patient esthetic and functional outcomes. Moreover, bone regenerative procedures by means of onlay autogenous bone graft and maxillary sinus augmentation allowed implant placement in an ideal tridimensional position, which favored the manufacturing of a proper prosthesis regarding function, esthetics, and biological properties, known as periodontal prosthesis.

## Data Availability

The data used to support the findings of this study are available from the corresponding author upon request.
